# The Application of Curve Fitting on the Voltammograms of Various Isoforms of Metallothioneins–Metal Complexes

**DOI:** 10.3390/ijms18030610

**Published:** 2017-03-11

**Authors:** Miguel Angel Merlos Rodrigo, Jorge Molina-López, Ana Maria Jimenez Jimenez, Elena Planells Del Pozo, Pavlina Adam, Tomas Eckschlager, Ondrej Zitka, Lukas Richtera, Vojtech Adam

**Affiliations:** 1 Department of Chemistry and Biochemistry, Mendel University in Brno, Zemedelska 1, CZ-613 00 Brno, Czech Republic; merlos19792003@hotmail.com (M.A.M.R.); anuskajj@hotmail.com (A.M.J.J.); Pavlina.Adam@mze.cz (P.A.); ZitkaO@seznam.cz (O.Z.); oliver@centrum.cz (L.R.); 2 Central European Institute of Technology, Brno University of Technology, Purkynova 123, CZ-612 00 Brno, Czech Republic; 3 Department of Physiology, Institute of Nutrition and Food Technology, University of Granada, Avenida Del Conocimiento S/N Biomedical Research Centre, Health Campus, 18001 Granada, Spain; jrgmolinalopez@ugr.es (J.M.-L.); elenamp@ugr.es (E.P.d.P.); 4 Department of Paediatric Haematology and Oncology, 2nd Faculty of Medicine, Charles University and University Hospital Motol, V Uvalu 84, CZ-150 06 Prague 5, Czech Republic; Tomas.Eckschlager@fnmotol.cz

**Keywords:** electrochemistry, mass spectrometry MALDI-TOF, metallothionein, metallomics, signal resolving

## Abstract

The translation of metallothioneins (MTs) is one of the defense strategies by which organisms protect themselves from metal-induced toxicity. MTs belong to a family of proteins comprising MT-1, MT-2, MT-3, and MT-4 classes, with multiple isoforms within each class. The main aim of this study was to determine the behavior of MT in dependence on various externally modelled environments, using electrochemistry. In our study, the mass distribution of MTs was characterized using MALDI-TOF. After that, adsorptive transfer stripping technique with differential pulse voltammetry was selected for optimization of electrochemical detection of MTs with regard to accumulation time and pH effects. Our results show that utilization of 0.5 M NaCl, pH 6.4, as the supporting electrolyte provides a highly complicated fingerprint, showing a number of non-resolved voltammograms. Hence, we further resolved the voltammograms exhibiting the broad and overlapping signals using curve fitting. The separated signals were assigned to the electrochemical responses of several MT complexes with zinc(II), cadmium(II), and copper(II), respectively. Our results show that electrochemistry could serve as a great tool for metalloproteomic applications to determine the ratio of metal ion bonds within the target protein structure, however, it provides highly complicated signals, which require further resolution using a proper statistical method, such as curve fitting.

## 1. Introduction

### 1.1. Metallothioneins

Ubiquitous metallothioneins (MTs) are relatively small metalloproteins, which are characterized by their high cysteine content (together with the absence of aromatic amino acids), and by their high affinity to a wide range of metal ions [1]. The first mammalian MTs were discovered by Margoshes and Vallée in 1957 [2]. During the time of research on these proteins, it has been revealed that they are involved in a number of substantial physiological phenomena, as they maintain essential metal (e.g., zinc) homeostasis, scavenge reactive oxygen species, regulate gene expression, and contribute to tissue regeneration [3,4,5,6]. Mammalian MTs’ structure consists of two zinc(II) clusters: Zn_4_Cys_11_ (α domain) and Zn_3_Cys_9_ (β domain). These domains play a crucial role in Zn^2+^ ion storage and donation to other metalloproteins, and they are implicated in several diseases [7]. MTs belong to a family of proteins with molecular weights of around 6000 Da, comprising MT-1, MT-2, MT-3, and MT-4 classes with multiple isoforms within each class. Two major metabolic factors are associated with zinc(II) metabolism: MT-1 and zinc(II) transporter 1 (ZnT-1). The relationship between maternal zinc(II) deficiency and significant decreases in placental MT-1 and ZnT-1 mRNA expression was observed in case of fetal heart malformations. Expression of ZnT-1 mRNA below the threshold levels may be a crucial factor to early determination of fetal heart malformations [8]. Several studies reported a modulatory effect of MT-3 and zinc(II) content on autophagic vacuole formation and lysosomal changes in cultured brain cells [9,10]. Different analytic and spectroscopic techniques can be used for determination and characterization of metal–MT complexes [11,12,13,14,15], however, MT complexation of metal ions is still of interest. Here, we show a simple method for characterization of metal–MT complexes in three selected mammalian MT isoforms (human and rabbit) by using an electrochemical technique with subsequent demonstration of statistical resolving of obtained voltammetric signals.

### 1.2. Evaluation of Non-Resolved Voltammetric Signals

There are many ways to process electrochemical signals. The development of new methods for electrochemical signal processing and the current state of these topics are clearly discussed and summarized in the review by Jakubowska et al. [16]. Among others, methods for baseline correction and methods for extracting more information from non-resolved voltammograms or voltammograms with overlapping signals attract much attention [17,18,19]. In the case of baseline, a straight line is conventionally used to evaluate peak height or peak area. If the evaluated peak is not on the flat part of the voltammogram, the straight line does not fit the real baseline and evaluation using this simplified approach leads to errors [20]. If an evaluated peak is located on the rising or descending part of the voltammogram, the peak height or area could be very significantly underestimated. In the case of lower analyte concentration, the evaluation of small peak intensities leads to high inaccuracy and to relatively large errors [21]. An analogous situation occurs for the appropriate evaluation methodology of overlapping signals. This problem arises when reduction energies of some electrochemical processes are close, and its significance is especially evident in the case when the voltammetric signals have high concentration ratios. Experimentally, it is possible to solve this problem through the use of some separation techniques, complexometric methods, and experimental optimization such as pH changes, supporting electrolyte, and the use of modified electrodes [16]. Iterative subtraction of the curve recorded for the one pure constituent of a mixture is another eventuality. Despite these possibilities, signal processing using numerical algorithms is most often used in the resolution of overlapping signals. These methods typically include a curve fitting, Fourier deconvolution, or wavelet based algorithms. The Fourier self-deconvolution technique is based on the multiplication of the Fourier transformation of the original signals by the weighting function in the frequency domain, which decays more slowly, and then the transformation of the multiplied signal back to the time domain [22]. This approach has already been successfully when used for easier evaluation of many electrochemical signals, such as for separation of voltammetric signals of Cd^2+^ and Tl^+^ [22,23] or in the case of simultaneous determination of Fe^2+^ and Mn^2+^ [24]. It has also been used for enhancing the separation yields of electrochemical signals of DNA nucleotides determined by square wave voltammetry [25]. The use of deconvolution in the case of poorly resolved or non-resolved signals increases separation efficiency and the sensitivity, leading to improved limits of detection and quantification [26,27,28,29,30,31]. Satisfactory effects of this approach were found even in the case of deconvolution of MT voltammograms [32]. The curve-fitting method used in our work is based on the simulation of a complex signal as the sum of single peak models, using iterative least-squares minimization. As in the case of deconvolution, the curve-fitting method has been used successfully in previously published studies [33,34,35].

## 2. Results and Discussion

### 2.1. Characterization of MTs by matrix-assisted laser desorption/ionization time-of-flight mass spectrometry (MALDI-TOF MS)

The matrix consists of small organic compounds, which show strong resonance absorption at the applied laser wavelength. In the majority of the studies reviewed, 2,5-dihydroxybenzoic acid (DHB) and α-cyano-4-hydroxycinnamic acid (HCCA) were the constituents of matrix used for appropriate determination of MTs [36,37]. In our study, DHB exhibited a significant rabbit MT2 protein (rMT2) concentration-dependent increase of observed signal intensity (a.u.) compared to HCCA (Figure S2A1). Utilization of both matrixes resulted in the different types of crystals. HCCA produced uniform crystals (Figure S2A2), while DHB produced heterogeneous and robust crystals (Figure S2A3). Further, to verify a proper heterologous expression of human MTs (hMTs) isolated from *Escherichia coli*, we analyzed their mass distribution. It is evident that Apo-MTs were not present. All MTs were expressed in heterologous organisms, so that they appear to form chelation or binding to other proteins, metals, or compounds in the bacteria, culture medium, and buffers. The main observed signals for hMTs shown in (Figure S2) were quasimolecular ions assigned as follows: [rMT2]^+^ (*m*/*z* 6210.94) (Figure S2A1), [6× His-tag-hMT2A]^+^ (*m*/*z* 7278.29) (Figure S2B) (corresponding to matrix cluster with 6× His-tag, the hMT2A protein has an added 1 kDa of molecular weight), and [hMT3]^+^ (*m*/*z* 6907.37) (Figure S2C).

### 2.2. Optimization of Electrochemical Detection for Determination of MT by Using NaCl as Supporting Electrolyte

A characterization of metal–MT complexes by using electrochemical methods, specifically by differential pulse voltammetry (DPV), was the main aim of this study. The influence of individual MT isoforms on electrochemical response was the main tested parameter. Electrochemical analyses were carried out in the presence of 0.5 M NaCl as a supporting electrolyte. Previous studies showed that MTs analyzed by using DPV provides a number of electrochemical signals assigned as: MT(Cd), MT(Cu), CdT’, ZnT’, CdT, and ZnT, which belong to various complexes of MTs with metal ions present in the analyzed solution [38,39,40]. Together with abovementioned signals, the voltammograms can contain the redox signals of free ions of Cd^2+^ and Zn^2+^ [38,39,40]. In such a case, the presence of MTs is confirmed by an evaluation of selected peaks, named as follows: ZnT, CdT, and MT(Cu).

First, our main interests were focused on obtaining the best possible analytical conditions for detection of individual MT isoforms. Hence, we have monitored three parameters, which are expected to significantly influence the analytical performance: (i) accumulation time (120, 240, and 360 s); (ii) concentration of supporting electrolyte (0.1, 0.3, and 0.5 M); and (iii) pH (6.5, 7.0, and7.5). In case of hMT2A protein, the CdT peak was not found in the electrochemical record. Insignificant potential changes of individual peaks were observed by using different conditions.

**Figure 1:**
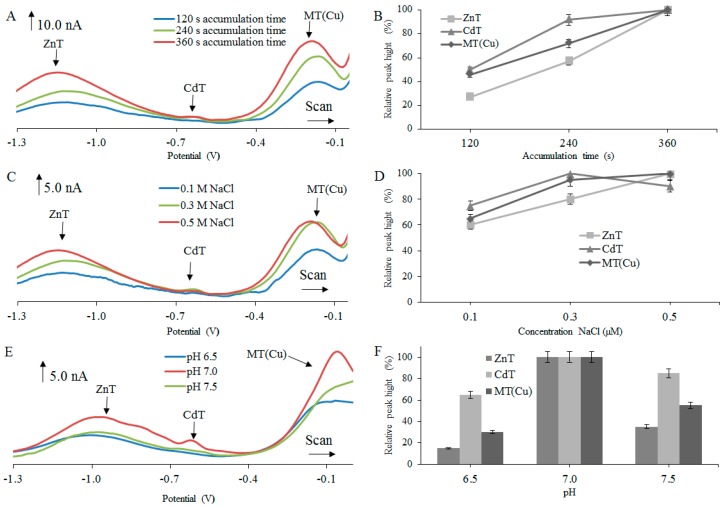
Differential pulse (DP) voltammograms of 2 µM rabbit MT2 protein (rMT2) measured in different (**A**) accumulation times, (**C**) concentration of NaCl, and (**E**) pH. Adsorptive transfer stripping technique coupled with differential pulse voltammetry (AdTS DPV) parameters were as follows: initial potential −1.5 V, end potential 0.0 V, modulation time 0.057 s, time interval 0.2 s, step potential of 1.05 mV/s, and modulation amplitude of 25 mV. Dependences of heights of ZnT, CdT, and MT(Cu) signals on (**B**) accumulation times of 120 , 240 , 360 s; (**D**) concentration of electrolyte (0.1, 0.3, 0.5 M); and (**F**) pH (6.5, 7.0, 7.5), expressed in relative percentage peaks and peak height (nA).

**Figure 2:**
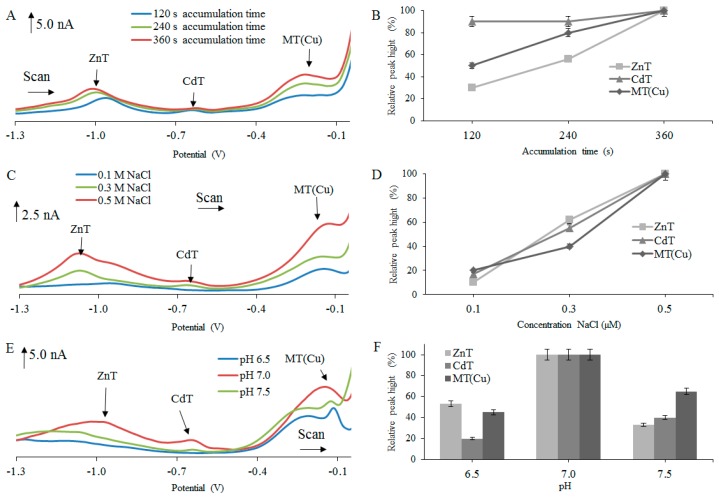
DP voltammograms of 2 µM human MT3 protein (hMT3) from brain measured in different (**A**) accumulation times, (**C**) concentration of NaCl, and (**E**) pH. AdTS DPV parameters were as follows: initial potential −1.5 V, end potential 0.0 V, modulation time 0.057 s, time interval 0.2 s, step potential of 1.05 mV/s, modulation amplitude of 25 mV. Dependences of heights of ZnT, CdT, and MT(Cu) signals on (**B**) accumulation times of 120, 240, 360 s; (**D**) concentration of electrolyte (0.1, 0.3, 0.5 M); and (**F**) pH (6.5, 7.0, 7.5), expressed in relative percentage peaks and peak height (nA).

**Figure 3:**
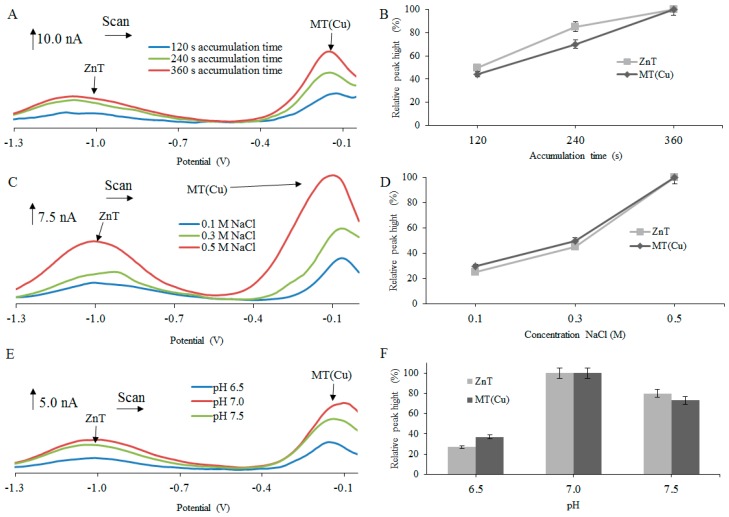
DP voltammograms of 2 µM human MT2A protein (hMT2A) from blood measured in different (**A**) accumulation times, (**C**) concentration of NaCl, and (**E**) pH. AdTS DPV parameters were as follows: initial potential −1.5 V, end potential 0.0 V, modulation time 0.057 s, time interval 0.2 s, step potential of 1.05 mV/s, modulation amplitude of 25 mV. Dependences of heights of ZnT and MT(Cu) signals on (**B**) accumulation times of 120, 240, 360 s; (**D**) concentration of electrolyte (0.1, 0.3, 0.5 M); and (**F**) pH (6.5, 7.0, 7.5), expressed in relative percentage peaks and peak height (nA).

The experimental results revealed that the electrochemical signals, observed after testing various isoforms of MT, increased with increasing accumulation time in the range from 120 to 360 s (Figure 1A,B, Figure 2A,B and Figure 3A,B). The biggest increases of the detected signals were observed for ZnT peaks, particularly in the case of rMT2 (Figure 1B), where about a 73% increase of the ZnT peak was found, and in the case of rMT3 where about a 70% increase of the ZnT peak was determined (Figure 2B). Therefore, for the next experiments, the accumulation time of 360 s was chosen as optimal.

The intensity of the electrochemical signal was evaluated with respect to the concentration of supporting electrolyte. In this case, the NaCl concentration gradually increased in the range from 0.1 to 0.5 M. The signals with the highest intensity were obtained at 0.5 M NaCl (Figure 1C,D, Figure 2C,D and Figure 3C,D). The biggest change of signal was observed in hMT2A (Figure 3D) at the peak of ZnT (75%) and at the peak MT(Cu) (70%). Quite interesting is the influence of NaCl concentration on obtained signals for hMT2A. Here, the differences among signal intensities at concentrations 0.3 M and 0.5 M were more significant, in comparison with the two other MTs.

The influence of the supporting electrolyte’s pH on the electrochemical signal intensity was another investigated parameter. Tested pH range was set from 6.5 to 7.5. For pH adjustment, 10 mM hydrochloric acid was used. From Figure 1E,F, Figure 2E,F and Figure 3E,F, it is clear that the highest electrochemical signals of individual peaks were obtained at pH 7.0. In the case of hMT2A (Figure 3E,F), changes in pH from 6.5 to 7.0 led to the increase of signal intensity of the ZnT peak of about 63% and the MT(Cu) peak of about 73%. In case of hMT3 (Figure 2E,F) there was an increase of the CdT peak intensities of about 80%. The rMT2 (Figure 1E,F) showed an increase in signal intensity of the ZnT peak of about 85%. For further experiments, optimal parameters that provide the highest and most stable electrochemical signal have been selected. In the case of the time accumulation, the value 360 s was selected. As the optimal electrolyte, a solution of 0.5 M NaCl at pH 7.0 was selected for all three MT forms.

All performed measurements provided non-resolved voltammograms with broad signals. The character of measured voltammetric curves, however, in almost all cases, exhibited the presence of more than three basic signals, discussed and evaluated in Figure 1, Figure 2 and Figure 3. It is obvious that voltammetric curves are the result of superposition of electrochemical signals which are very close to each other and which very probably belong to different MT complexes with different metal ions. To distinguish each of these strongly overlapping signals and to obtain separated peak heights and positions, the curve-fitting method was employed, as it is further described.

### 2.3. The Application of Curve Fitting on Non-Resolved Voltammetric Signals of Metal-MT Complexes

**Figure 4:**
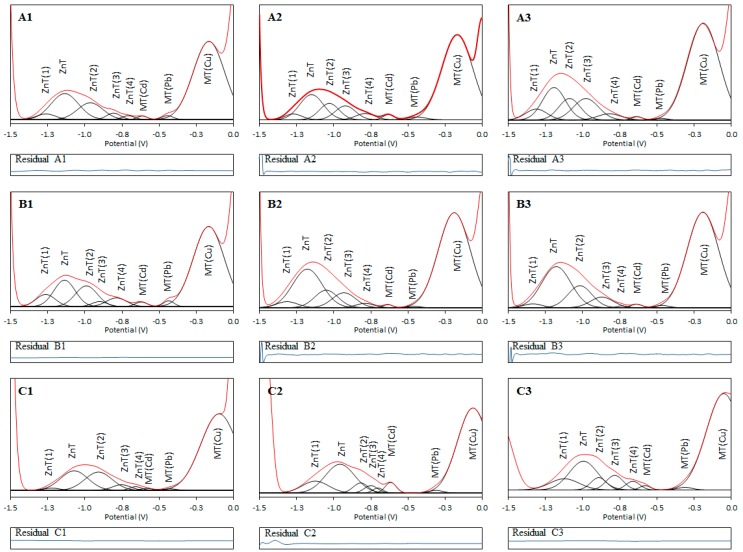
Resolved voltammograms of rMT2 after application of curve fitting method. (**A1**–**A3**) Accumulation times: 120, 240, and 360 s; (**B1**–**B3**) concentration of NaCl: 0.1, 0.3, and 0.5 M; (**C1**–**C3**) pH: 6.5, 7.0, and 7.5. Individual voltammetric signals of metal–MT complexes (black lines), measured non-resolved voltammetric signals (red line), residuals (blue line).

In analyzed rMT2, hMT2A and hMT3 voltammograms, we observed two or three relatively broad signals which had a quite high half-width value and whose shape did not correspond to a simple electrochemical signal. An attempt to evaluate the maxima of these non-resolved peaks is shown in Figure 1, Figure 2 and Figure 3; the assignment of signals to MT complexes with Zn^2+^, Cd^2+^, and Cu^2+^ was performed on the basis of the work in [41]. A broad signal in the region from −1.1 to −1.0 V was assigned to ZnT, the signal at −0.65 V was labeled as MT(Cd), and the signal in the range from −0.3 to −0.1 V was assigned to MT(Cu). More details about the assignment and origin of these signals can be found in some previous works [38,39,40,42,43]. From the nature of all signals, it is clear that these signals are non-resolved signals that indicate the existence of more species or forms with similar reduction energies and therefore similar electrochemical properties [16]. To determine each signal’s exact position and possible intensity, the curve-fitting method for the whole voltammogram range was performed (Figure 4 illustrates the deconvoluted voltammograms for rMT2, other deconvoluted voltammograms are in Figures S3 and S4). Detailed results of the curve-fitting method for each separated peak can be found in Supplementary Materials (Tables S1–S3). Before applying the curve-fitting method to each voltammogram, a subtraction of the linear background was performed. The curve-fitting method was performed using simple Gaussian curves; the number of hidden signals was derived by careful analysis of all MT curve profiles of individual isoforms, so that for the fitting the minimum possible number of signals was applied. In each case, the “fit sum plot” resulting model exhibited perfect conformity based on the minimal “residual” (difference between measured voltammogram curve and fitted model). Suburb parts of voltammograms were processed using the auxiliary peaks (also Gaussian curve), which are omitted in the final visualization for clarity.

**Figure 5:**
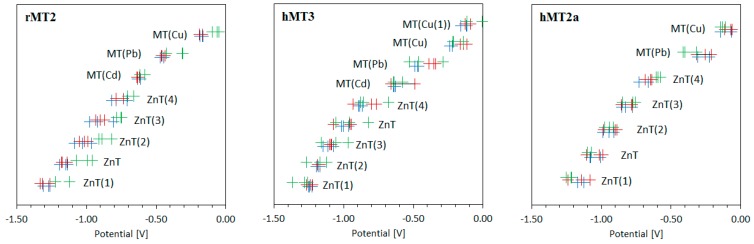
Deconvoluted peak potential for rMT2, hMT3, and hMT2a for accumulation (+++), concentration (+++), and pH (+++).

Found positions of individual resolved signals of different metal complexes of individual MT isoforms are summarized in Figure 5 and Tables S1–S3. In accordance with expectations, it can be stated that the change in concentration has a more significant impact on the peak height than on its position, while the influence of pH is reflected by noticeable changes in the positions of the resolved signals (Figure 4, Figures S3 and S4).

## 3. Materials and Methods

### 3.1. Reagents and Chemicals

Chemicals were obtained from Sigma-Aldrich (St. Louis, MO, USA) in ACS purity unless noted otherwise. In this study, high-purity deionized water (Milli-Q Millipore 18.2 MΩ/cm, Bedford, MA, USA) was used. Water was prepared using reverse osmosis equipment Aqual 25 (Aqual, Brno, Czech Republic) and further purified by using apparatus Milli-Q Direct QUV equipped with the UV lamp (Milli-Q water, 18 MΩ, Millipore Corp., Billerica, MA, USA). WTW inoLab pH meter (Weilheim, Germany) was used for pH measurement.

### 3.2. Isolation of MTs and Construction of Plasmids for Heterologous Expression of MTs

The human MT3 protein (hMT3) was cloned in the pRSET-B vector (Invitrogen, Waltham, MA, USA). The construction of human MT3–pRSET-B plasmid (Figure S1A) was obtained at Faculty of Science, Masaryk University, Brno, Czech Republic. The chemical transformation protocol was performed following the instructions of New England Biolabs (Ipswich, MA, USA), using BL21(DE3)pLysS chemically competent *Escherichia coli* as a host. Bacteria transformed with hMT3–pRSET-B plasmid were selected by their ampicillin resistance.

The *hMT2A* gene was identified in the NCBI database (Reference Sequence: NM_005953.3) (Figure S1B) and isolated (MagNA Pure Compact Nucleic Acid Isolation Kit I, Roche, Indianapolis, IN, USA) from fresh blood donated by a healthy human donor. Subsequent PCR amplification used a set of primers flanking the complete open reading frame from 5′ and 3′ untranslated regions. The primers were as follows: 5′-CAACCTGTCCCGACTCTAGC-3′ (*hMT2A*fw) and 5′-TTGTGGAAGTCGCGTTCTTT-3′ (*hMT2A*rev). The orientation of the *hMT2A* sequence isolated from the blood samples within the cloning vector was properly checked by sequencing (Promega, Madison, WI, USA) (Figure S1C). For expression, *hMT2A* gene was subcloned into the pRSET-B vector (Figure S1D).

The rabbit MT2 protein (rMT2) was isolated from the liver of CdCl_2_-administered rabbits according to our previous study [44]. Sample prepared like this was used for isolation of rMT2 using fast protein liquid chromatography (FPLC).

### 3.3. The Matrix-Assisted Laser Desorption/Ionization Time-of-Flight Mass Spectrometry (MALDI-TOF MS)

MTs were purified by using FPLC according to our previous study [44]. After isolation, MTs were analyzed using MALDI-TOF MS (Ultraflex III instrument, Bruker Daltonik, Leipzig, Germany) equipped with a laser, operating at wavelength of 355 nm with an accelerating voltage of 25 kV, a maximum energy of 43.2 µJ, and a repetition rate of 2000 Hz. The matrixes used for analyses were α-cyano-4-hydroxycinnamic acid (HCCA) and 2,5-dihydroxybenzoic acid (DHB) (Bruker Daltonik, Leipzig, Germany) prepared in acetonitrile solution (30% *w*/*w*) with the addition of trifluoroacetic acid (0.1% *w*/*w*). Matrix and substance solutions for analysis were mixed in ratio of 1:1 (*v*/*v*). Obtained homogeneous solution (1 µL) was dried under atmospheric pressure and ambient temperature (25 °C). MS spectra were typically acquired by averaging 20 sub-spectra from a total of 500 shots of the laser (Smartbeam 2. Version: 1_0_38.5, Bruker Daltonik, Leipzig, Germany).

### 3.4. Electrochemical Measurements

Autolab Analyzer (EcoChemie, Utrecht, Netherlands) connected to VA-Stand 663 (Metrohm G.A, Herissau, Switzerland) was used for electrochemical measurements. A standard cell with three-electrode system was used for all experiments, with a hanging mercury drop electrode as the working electrode (drop area of 0.4 mm^2^); a Ag/AgCl/3 M KCl and a platinum electrode were used as a reference and an auxiliary electrode, respectively. MTs were measured using adsorptive transfer stripping technique coupled with differential pulse voltammetry (AdTS DPV). As the supporting electrolyte, sodium chloride (0.5 M NaCl, pH 6.4) was used. DPV parameters were as follow: the initial potential of −1.5 V, the end potential 0.0 V, modulation time 0.057 s, time interval 0.2 s, step potential 1.05 mV·s^−1^, modulation amplitude 25 mV. Calibration curve was measured in acetate buffer, pH 5. All experiments were carried out at ambient temperature (25 °C).

### 3.5. Software Used for Data Collection and Evaluation

GPES 4.9 software (supplied by EcoChemie) was used for data collection. Data obtained were further analyzed and processed using eL-Chem Viewer 2.1 (L-Chem, Horka nad Moravou, Czech Republic) [45]. For curve-fitting method, MagicPlot software (developed by Magicplot Systems, LLC, Saint Petersburg, Russia) was used. Subsequently, where possible, data were graphically and mathematically processed using Microsoft Excel^®^ and Microsoft PowerPoint^®^.

## 4. Conclusions

Electrochemical methods that use heavy-metal-binding by MTs and other peptides or proteins as a biological portion belong to a set of interesting tools for sensitive analysis of their interaction. Data presented in this work highlight the potential of DPV to be used for monitoring differences in behavior of various MT isoforms and their complexes with metals. The effect of some metals bound in the structures of MTs has been studied several times, and not only DPV has been used for this purpose [39,46,47,48]. From these studies, it can be concluded that there are differences in the specificity, capacity, and dissociation constants among metals. Palladium and platinum and their compounds belong to a class that binds to MT with the highest affinity [46,47]. There have also been some interesting structural changes observed in the protein structure due to metal binding [49,50,51].

Additionally, the curve-fitting method was applied to the non-resolved DPV voltammograms of all used MT isoforms, and individual peaks were separated. The influence of different conditions (accumulation time, concentration, and pH) on peak height and peak intensity was observed. Our data indicate that although electrochemistry can be suitable and sufficiently sensitive to specify the complexation of metals in MT isoforms, additional data processing is required to obtain further insight into this phenomenon, which can be highly interesting for understanding the biological effects of these complexes.

## Supplementary Materials

Supplementary materials can be found at www.mdpi.com/1422-0067/18/3/610/s1.
