# Supplementary material for: The Application of Curve Fitting on the Voltammograms of Various Isoforms of Metallothioneins–Metal Complexes
Source: Int J Mol Sci. 2017 Mar 11;18(3):610. doi: 10.3390/ijms18030610 (PMC5372626; doi:10.3390/ijms18030610)
Supplement: Supplementary file 1 [file ijms-18-00610-s001.pdf]

Supplementary data

The application of curve fitting on the voltammograms of various isoforms of metallothioneins-metal complexes

Miguel Angel Merlos Rodrigo [1,2], Jorge Molina-López [3], Ana Maria Jimenez Jimenez [1,2], Elena Planells [3], Pavlina Adam [1,2], Tomas Eckschlager [4], Ondrej Zitka [1,2], Lukas Richtera [1,2], Vojtech Adam [1,2,*]

1. Department of Chemistry and Biochemistry, Mendel University in Brno, Zemedelska 1, CZ-613 00 Brno, Czech Republic; merlos19792003@hotmail.com (M.A.M.R.); anuskajj@hotmail.com (A.M.J.J.); Pavlina.Adam@mze.cz (P.A.); ZitkaO@seznam.cz (O.Z.); oliver@centrum.cz (L.R.)
2. Central European Institute of Technology, Brno University of Technology, Purkynova 123, CZ-612 00 Brno, Czech Republic
3. Department of Physiology, Institute of Nutrition and Food Technology, University of Granada, Avenida Del Conocimiento S/N Biomedical Research Centre, Health Campus, 18001 Granada, Spain; jrgmolinalopez@ugr.es (J.M.-L.); elenamp@ugr.es (E.P.P.)
4. Department of Paediatric Haematology and Oncology, 2nd Faculty of Medicine, Charles University, and University Hospital Motol, V Uvalu 84, Prague 5 CZ-15006, Czech Republic; Tomas.Eckschlager@fnmotol.cz (T.E.)

* Correspondence: vojtech.adam@mendelu.cz; Tel.: +420-5-4513-3350

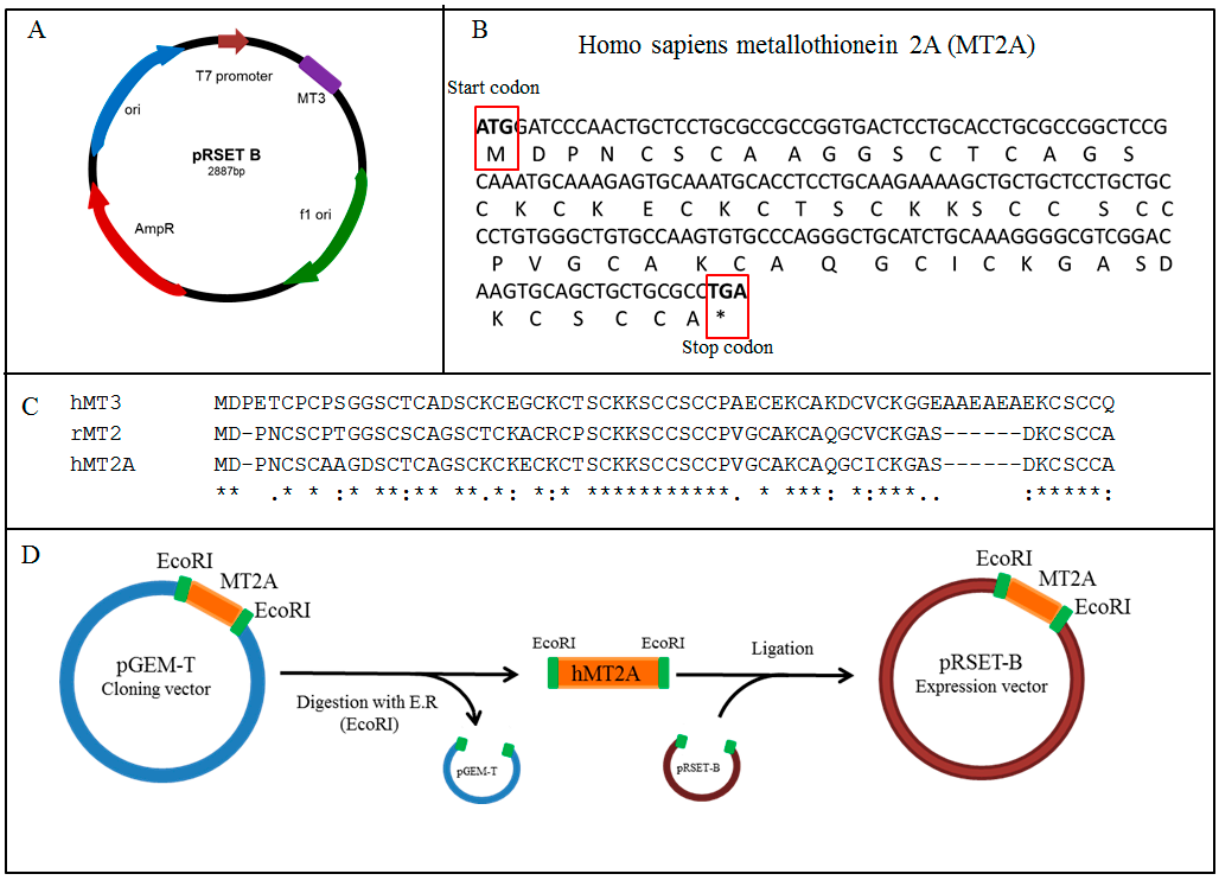

Figure S1. (**A**) The map upwards shows the features of hMT3-pRSET-B. (**B**) Nucleotide and amino acid sequences of human metallothionein 2A (*hMT2A*) were cloned in pRSET-B. Vector pRSET is control vector expressing β-galactosidase, which is fused to an *N*-terminal peptide containing the Xpress peptide, 6× His tag and an enterokinase recognition site. (**C**) Alignment of amino acid sequences of hMT3, rMT2 and hMT2A isoforms was performed using Multiple sequence alignment ClustalW2 algorithm available at EBI (http://www.ebi.ac.uk/Tools/clustalw2/). (**D**) Scheme of subcloning strategy for *hMT2A* from pGEM-T cloning vector to pRSET-B expression vector. EcoRI was the restriction enzyme used for isolation of the full-length gene from pGEM-T cloning vector. The chemical transformation protocol was performed following the instructions of New England Biolabs, using as host BL21(DE3)pLysS chemically competent Escherichia coli strain (for high level expression of recombinant protein). To obtain pRSET-hMT2A, the full-length hMT2A was isolated from the pGEM-T vector by digestion with restriction enzyme EcoRI and ligated in into the EcoRI-digested shuttle vector pRSET-B. The pRSET-B contains N-terminal polyhistidine tag (6× His-tag), to permits purification of recombinant fusion protein on metal-chelating resins. All plasmids were amplified by transformation of *E. coli* following standard procedures and purified by using the Qiagen Miniprep Kit (Qiagen, MD, USA). All positives transformants were confirmed by PCR screening (data not shown). The positive transformants of human MTs were grown in LB (Luria-Bertani) broth with 50 μg·mL^{-1} ampicillin and 35 μg·mL^{-1} chloramphenicol. The isolation protocol was performed following the instructions of pRSET A, B, and C for high-level expression of recombinant proteins in *E. coli* (Invitrogen, Waltham, MA, USA).

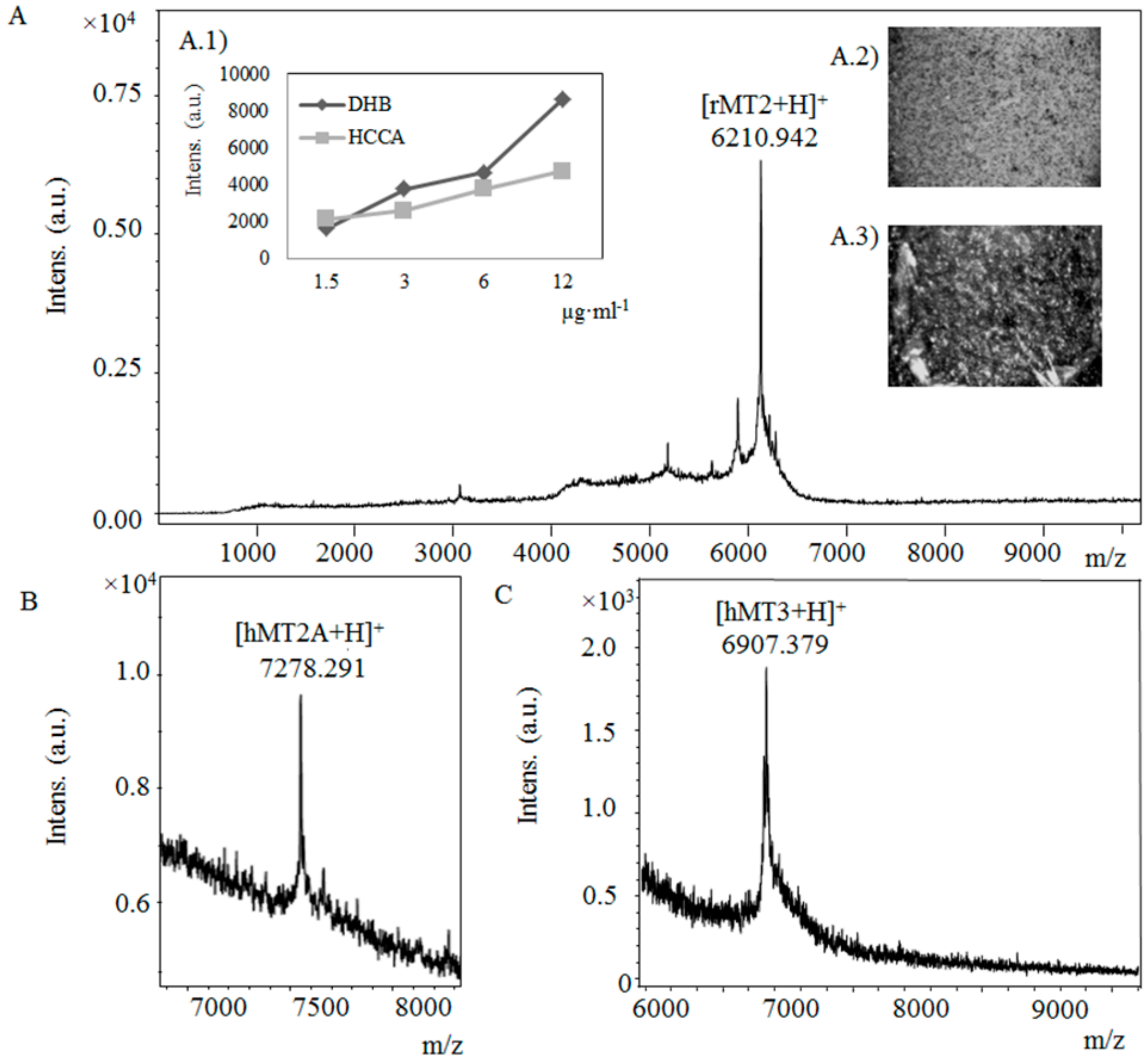

Figure S2. (**A.1**) Spectrum of rMT2 liver measured by MALDI-TOF MS with HCCA matrix and graphs of signal intensity of different concentrations of rMT2 in DHB and HCCA matrixes. Photo of the MTs crystals on a target plate using (**A.2**) DHB and (**A.3**) HCCA matrix. (**B**) Demonstrates spectrum of hMT2A with 6× His-tag and (**C**) hMT3 measured by using MALDI-TOF MS with DHB matrix prepared in TA30 at a maximum energy of 43.2 µJ with repetition rate 2000 Hz.

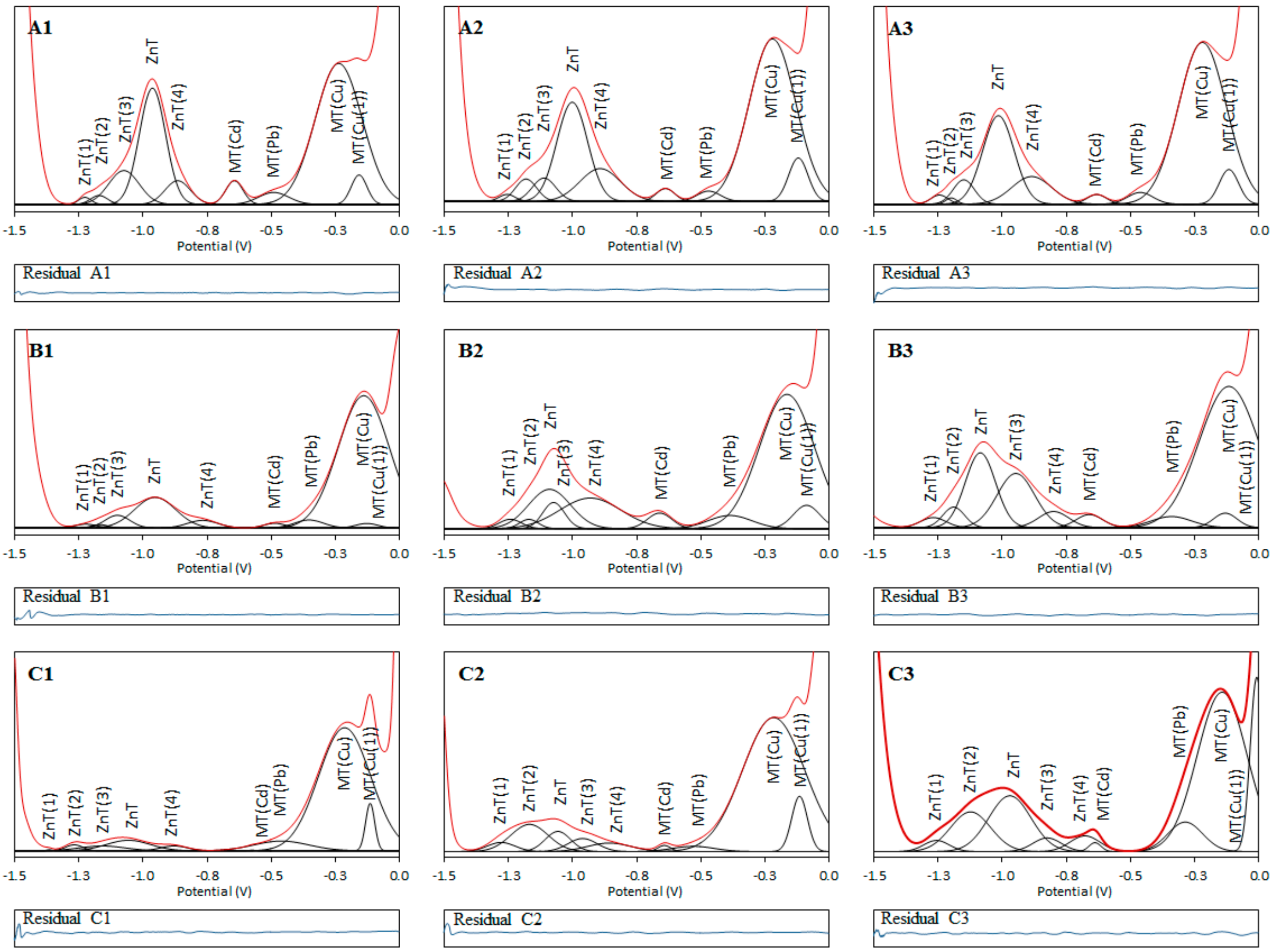

Figure S3. Resolved voltammograms of hMT3 after application of curve fitting method. (**A1-A3**): Accumulation times: 120 s, 240 s and 360 s. (**B1-B3**): concentration of NaCl: 0.1M, 0.3M and 0.5M. (**C1-C3**): pH: 6.5, 7.0 and 7.5. Individual voltammetric signals of metal-MTs complexes (black lines), measured non-resolved voltammetric signals (red line), residuals (blue line).

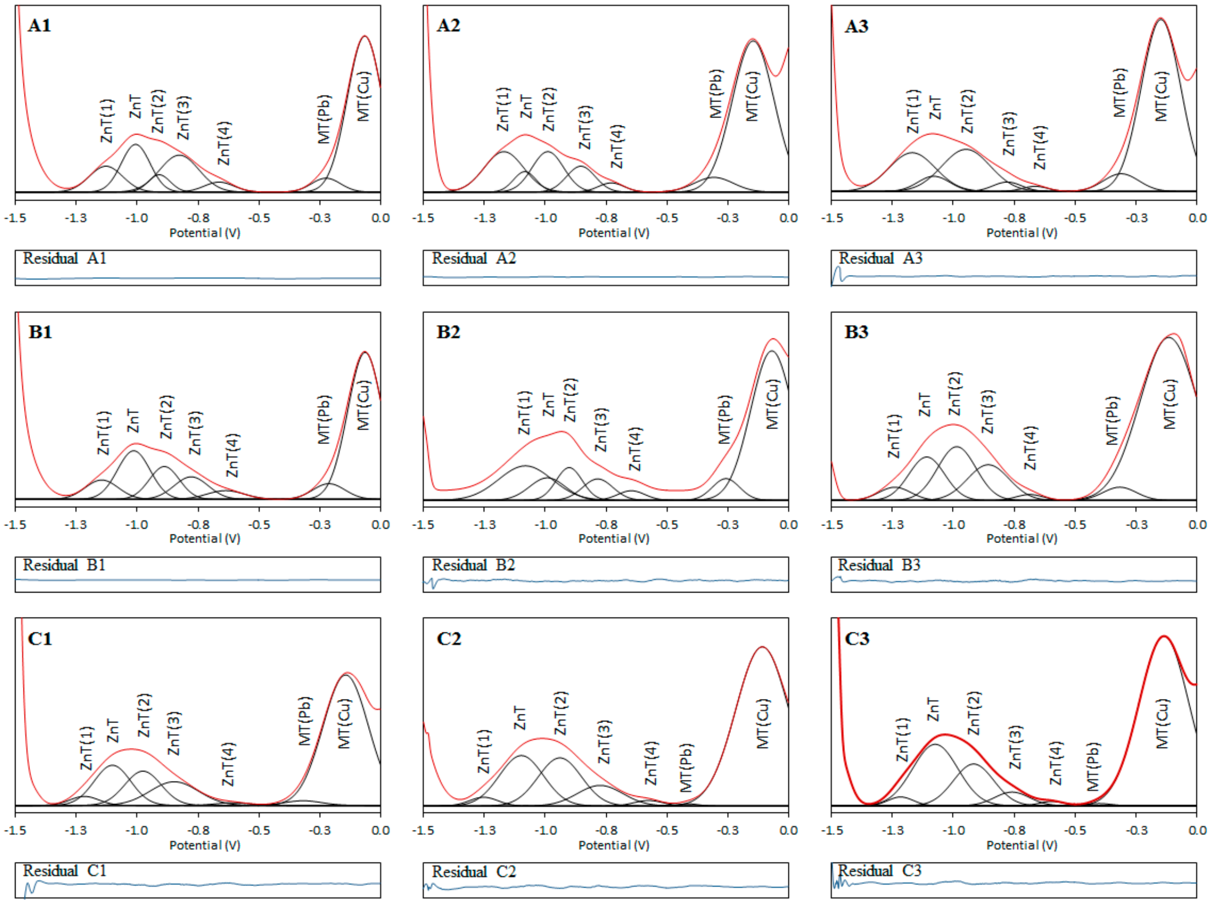

Figure S4. Resolved voltammograms of hMT2a after application of curve fitting method. (**A1–A3**): Accumulation times: 120 s, 240 s and 360 s. (**B1–B3**): Concentration of NaCl: 0.1M, 0.3M and 0.5M. (**C1–C3**): pH: 6.5, 7.0 and 7.5. Individual voltammetric signals of metal-MTs complexes (black lines), measured non-resolved voltammetric signals (red line), residuals (blue line).

Tables S1. List of individual peak parameters for rMT2 voltammograms (Fig 4. A1-C3).

General peak equation: $y(x) = \sqrt{\frac{\ln 2}{\pi}} \cdot \left(\frac{a}{d_x}\right) \cdot e^{\left(\frac{-\ln 2 \cdot (x - x_0)^2}{d_x^2}\right)}$.

Linear background correction equation: $y(x) = ax + b$.

A1 (background equation parameters parameters a = $-1.0650 \cdot 10^{-10}$; b = $-3.2542 \cdot 10^{-11}$)

peak	a (area)	x_0 (position)	d_x (HWHM)	ampl	s
ZnT(1)	$1.0328 \cdot 10^{-10}$	−1.2618	0.0757	$6.4119 \cdot 10^{-10}$	0.0643
ZnT	$6.8093 \cdot 10^{-10}$	−1.1357	0.1085	$2.9482 \cdot 10^{-9}$	0.0921
ZnT(2)	$4.0619 \cdot 10^{-10}$	−0.9639	0.1002	$1.9042 \cdot 10^{-9}$	0.0851
ZnT(3)	$8.6616 \cdot 10^{-11}$	−0.8075	0.0551	$7.3869 \cdot 10^{-10}$	0.0468
ZnT(4)	$4.6338 \cdot 10^{-11}$	−0.7078	0.0495	$4.3960 \cdot 10^{-10}$	0.0421
MT(Cd)	$3.4393 \cdot 10^{-11}$	−0.6137	0.0401	$4.0298 \cdot 10^{-10}$	0.0340
MT(Pb)	$4.2940 \cdot 10^{-11}$	−0.4439	0.0448	$4.5055 \cdot 10^{-10}$	0.0380
MT(Cu)	$2.3598 \cdot 10^{-9}$	−0.1655	0.1252	$8.8544 \cdot 10^{-9}$	0.1063
auxiliary peak (1)	$8.8114 \cdot 10^{-9}$	−1.6983	0.0842	$4.9148 \cdot 10^{-8}$	0.0715
auxiliary peak (2)	$2.3109 \cdot 10^{-7}$	−1.6897	0.0724	$1.4988 \cdot 10^{-6}$	0.0615
auxiliary peak (3)	$2.4207 \cdot 10^{-9}$	0.0413	0.0600	$1.8951 \cdot 10^{-8}$	0.0510

A2 (background equation parameters a = $-2.1957 \cdot 10^{-9}$; b = $-8.5994 \cdot 10^{-11}$)

peak	a (area)	x_0 (position)	d_x (HWHM)	ampl	s
ZnT(1)	$1.4096 \cdot 10^{-10}$	−1.2722	0.0687	$9.6387 \cdot 10^{-10}$	0.0583
ZnT	$8.3155 \cdot 10^{-10}$	−1.1493	0.0938	$4.1633 \cdot 10^{-9}$	0.0797
ZnT(2)	$4.4752 \cdot 10^{-10}$	−1.0297	0.0775	$2.7112 \cdot 10^{-9}$	0.0658
ZnT(3)	$3.9122 \cdot 10^{-10}$	−0.9219	0.0798	$2.3039 \cdot 10^{-9}$	0.0677
ZnT(4)	$1.6707 \cdot 10^{-10}$	−0.7879	0.0758	$1.0353 \cdot 10^{-9}$	0.0644
MT(Cd)	$7.9371 \cdot 10^{-11}$	−0.6320	0.0430	$8.6695 \cdot 10^{-10}$	0.0365
MT(Pb)	$7.3541 \cdot 10^{-11}$	−0.4433	0.0742	$4.6555 \cdot 10^{-10}$	0.0630
MT(Cu)	$3.6787 \cdot 10^{-9}$	−0.1688	0.1229	$1.4057 \cdot 10^{-8}$	0.1044
auxiliary peak (1)	$3.6539 \cdot 10^{-3}$	−1.8515	0.0781	$2.2000 \cdot 10^{-2}$	0.0663
auxiliary peak (2)	$6.7062 \cdot 10^{-10}$	−0.0083	0.0323	$9.7648 \cdot 10^{-9}$	0.0274
auxiliary peak (3)	$8.6632 \cdot 10^{-9}$	0.1324	0.0658	$6.1814 \cdot 10^{-8}$	0.0559

A3 (background equation parameters a = $-2.6072 \cdot 10^{-9}$; b = $5.5186 \cdot 10^{-11}$)

peak	a (area)	x_0 (position)	d_x (HWHM)	ampl	s
ZnT(1)	$3.4246 \cdot 10^{-10}$	−1.3112	0.0847	$1.9002 \cdot 10^{-9}$	0.0719
ZnT	$1.0865 \cdot 10^{-9}$	−1.1924	0.0904	$5.6434 \cdot 10^{-9}$	0.0768
ZnT(2)	$6.7723 \cdot 10^{-10}$	−1.0879	0.0852	$3.7322 \cdot 10^{-9}$	0.0724
ZnT(3)	$7.8999 \cdot 10^{-10}$	−0.9773	0.0995	$3.7307 \cdot 10^{-9}$	0.0845
ZnT(4)	$2.1153 \cdot 10^{-10}$	−0.8200	0.0922	$1.0773 \cdot 10^{-9}$	0.0783
MT(Cd)	$4.8870 \cdot 10^{-11}$	−0.6331	0.0408	$5.6272 \cdot 10^{-10}$	0.0346
MT(Pb)	$3.1263 \cdot 10^{-11}$	−0.4732	0.0507	$2.8955 \cdot 10^{-10}$	0.0431
MT(Cu)	$4.5350 \cdot 10^{-9}$	−0.1878	0.1268	$1.6804 \cdot 10^{-8}$	0.1077
auxiliary peak (1)	$5.2057 \cdot 10^{-9}$	−1.5394	0.0286	$8.5483 \cdot 10^{-8}$	0.0243
auxiliary peak (2)	$6.2893 \cdot 10^{-10}$	−0.0111	0.0407	$7.2496 \cdot 10^{-9}$	0.0346
auxiliary peak (3)	$5.3250 \cdot 10^{-8}$	0.0220	0.0978	$2.5569 \cdot 10^{-7}$	0.0831

B1 (background equation parameters a = $-3.7171 \cdot 10^{-11}$; b = $-2.7270 \cdot 10^{-11}$)

peak	a (area)	x_0 (position)	d_x (HWHM)	ampl	s
ZnT(1)	$2.2499 \cdot 10^{-10}$	−1.2640	0.0765	$1.3810 \cdot 10^{-9}$	0.0650
ZnT	$5.3747 \cdot 10^{-10}$	−1.1374	0.0841	$3.0006 \cdot 10^{-9}$	0.0715
ZnT(2)	$4.3601 \cdot 10^{-10}$	−0.9904	0.0871	$2.3512 \cdot 10^{-9}$	0.0740
ZnT(3)	$7.5438 \cdot 10^{-11}$	−0.9007	0.0619	$5.7247 \cdot 10^{-10}$	0.0526
ZnT(4)	$1.6785 \cdot 10^{-10}$	−0.7876	0.0799	$9.8719 \cdot 10^{-10}$	0.0678
MT(Cd)	$5.3703 \cdot 10^{-11}$	−0.6207	0.0482	$5.2372 \cdot 10^{-10}$	0.0409
MT(Pb)	$5.9007 \cdot 10^{-11}$	−0.4359	0.0421	$6.5849 \cdot 10^{-10}$	0.0357
MT(Cu)	$2.3714 \cdot 10^{-9}$	−0.1667	0.1227	$9.0762 \cdot 10^{-9}$	0.1042
auxiliary peak (1)	$2.3109 \cdot 10^{-7}$	−1.6897	0.0724	$1.4988 \cdot 10^{-6}$	0.0615
auxiliary peak (2)	$1.0280 \cdot 10^{-9}$	−1.5307	0.0190	$2.5381 \cdot 10^{-8}$	0.0162
auxiliary peak (3)	$1.8682 \cdot 10^{-9}$	0.0307	0.0521	$1.6843 \cdot 10^{-8}$	0.0442

B2 (background equation parameters a = $-2.0642 \cdot 10^{-9}$; b = $1.7367 \cdot 10^{-10}$)

peak	a (area)	x_0 (position)	d_x (HWHM)	ampl	s
ZnT(1)	$1.5585 \cdot 10^{-10}$	−1.3143	0.0833	$8.7832 \cdot 10^{-10}$	0.0708
ZnT	$1.4011 \cdot 10^{-9}$	−1.1773	0.1163	$5.6582 \cdot 10^{-9}$	0.0988
ZnT(2)	$5.0138 \cdot 10^{-10}$	−1.0503	0.0919	$2.5632 \cdot 10^{-9}$	0.0780
ZnT(3)	$4.2692 \cdot 10^{-10}$	−0.9311	0.0916	$2.1902 \cdot 10^{-9}$	0.0778
ZnT(4)	$9.5921 \cdot 10^{-11}$	−0.7889	0.0720	$6.2564 \cdot 10^{-10}$	0.0612
MT(Cd)	$3.4789 \cdot 10^{-11}$	−0.6357	0.0366	$4.4596 \cdot 10^{-10}$	0.0311
MT(Pb)	$1.9635 \cdot 10^{-11}$	−0.4569	0.0470	$1.9639 \cdot 10^{-10}$	0.0399
MT(Cu)	$3.7734 \cdot 10^{-9}$	−0.1890	0.1272	$1.3936 \cdot 10^{-8}$	0.1080
auxiliary peak (1)	$2.3109 \cdot 10^{-7}$	−1.5877	0.0324	$3.3479 \cdot 10^{-6}$	0.0275
auxiliary peak (2)	$1.4570 \cdot 10^{-9}$	−1.5457	0.0379	$1.8050 \cdot 10^{-8}$	0.0322
auxiliary peak (3)	$1.9160 \cdot 10^{-9}$	0.0182	0.0518	$1.7367 \cdot 10^{-8}$	0.0440

B3 (background equation parameters a = $-2.2577 \cdot 10^{-9}$; b = $5.3457 \cdot 10^{-12}$)

peak	a (area)	x_0 (position)	d_x (HWHM)	ampl	s
ZnT(1)	$8.5326 \cdot 10^{-11}$	−1.3347	0.0716	$5.5996 \cdot 10^{-10}$	0.0608
ZnT	$1.4785 \cdot 10^{-9}$	−1.1768	0.1154	$6.0196 \cdot 10^{-9}$	0.0980
ZnT(2)	$6.4890 \cdot 10^{-10}$	−1.0178	0.0945	$3.2245 \cdot 10^{-9}$	0.0803
ZnT(3)	$2.9885 \cdot 10^{-10}$	−0.8706	0.0908	$1.5460 \cdot 10^{-9}$	0.0771
ZnT(4)	$2.0109 \cdot 10^{-11}$	−0.7369	0.0371	$2.5447 \cdot 10^{-10}$	0.0315
MT(Cd)	$4.7389 \cdot 10^{-11}$	−0.6372	0.0441	$5.0517 \cdot 10^{-10}$	0.0374
MT(Pb)	$3.6718 \cdot 10^{-11}$	−0.4639	0.0530	$3.2513 \cdot 10^{-10}$	0.0451
MT(Cu)	$3.7449 \cdot 10^{-9}$	−0.1905	0.1257	$1.3995 \cdot 10^{-8}$	0.1068
auxiliary peak (1)	$2.3109 \cdot 10^{-7}$	−1.5782	0.0294	$3.6893 \cdot 10^{-6}$	0.0250
auxiliary peak (2)	$7.5992 \cdot 10^{-10}$	−1.5263	0.0285	$1.2507 \cdot 10^{-8}$	0.0242
auxiliary peak (3)	$1.3016 \cdot 10^{-9}$	0.0082	0.0466	$1.3113 \cdot 10^{-8}$	0.0396
auxiliary peak (4)	$7.1615 \cdot 10^{-9}$	0.2155	0.1277	$2.6350 \cdot 10^{-8}$	0.1084

C1 (background equation parameters a = $2.4178 \cdot 10^{-11}$; b = $2.8748 \cdot 10^{-11}$)

peak	a (area)	x_0 (position)	d_x (HWHM)	ampl	s
ZnT(1)	$2.1918 \cdot 10^{-11}$	−1.2220	0.0634	$1.6249 \cdot 10^{-10}$	0.0538
ZnT	$3.2533 \cdot 10^{-10}$	−1.0721	0.1094	$1.3972 \cdot 10^{-9}$	0.0929
ZnT(2)	$2.9982 \cdot 10^{-10}$	−0.9088	0.1079	$1.3054 \cdot 10^{-9}$	0.0916
ZnT(3)	$6.2430 \cdot 10^{-11}$	−0.7542	0.0727	$4.0321 \cdot 10^{-10}$	0.0618
ZnT(4)	$2.7150 \cdot 10^{-11}$	−0.6583	0.0488	$2.6119 \cdot 10^{-10}$	0.0415
MT(Cd)	$1.4847 \cdot 10^{-11}$	−0.5830	0.0500	$1.3948 \cdot 10^{-10}$	0.0425
MT(Pb)	$1.3047 \cdot 10^{-11}$	−0.4286	0.0553	$1.1073 \cdot 10^{-10}$	0.0470
MT(Cu)	$1.6849 \cdot 10^{-9}$	−0.0970	0.1450	$5.4586 \cdot 10^{-9}$	0.1231
auxiliary peak (1)	$2.3109 \cdot 10^{-7}$	−1.6700	0.0724	$1.4988 \cdot 10^{-6}$	0.0615
auxiliary peak (2)	$9.1970 \cdot 10^{-10}$	0.0111	0.0334	$1.2936 \cdot 10^{-8}$	0.0284

C2 (background equation parameters a = $-2.1195 \cdot 10^{-12}$; b = $-4.5057 \cdot 10^{-12}$)

peak	a (area)	x_0 (position)	d_x (HWHM)	ampl	s
ZnT(1)	$2.9952 \cdot 10^{-10}$	−1.1240	0.1049	$1.3410 \cdot 10^{-9}$	0.0891
ZnT	$8.0538 \cdot 10^{-10}$	−0.9578	0.1162	$3.2559 \cdot 10^{-9}$	0.0987
ZnT(2)	$1.4283 \cdot 10^{-10}$	−0.8183	0.0589	$1.1390 \cdot 10^{-9}$	0.0500
ZnT(3)	$9.3866 \cdot 10^{-11}$	−0.7499	0.0527	$8.3726 \cdot 10^{-10}$	0.0447
ZnT(4)	$4.7369 \cdot 10^{-11}$	−0.7025	0.0396	$5.6172 \cdot 10^{-10}$	0.0336
MT(Cd)	$1.1128 \cdot 10^{-10}$	−0.6168	0.0437	$1.1973 \cdot 10^{-9}$	0.0371
MT(Pb)	$3.4391 \cdot 10^{-11}$	−0.3114	0.0545	$2.9628 \cdot 10^{-10}$	0.0463
MT(Cu)	$2.5451 \cdot 10^{-9}$	−0.0628	0.1242	$9.6245 \cdot 10^{-9}$	0.1055
auxiliary peak (1)	$3.1425 \cdot 10^{-9}$	−1.5209	0.0625	$2.3618 \cdot 10^{-8}$	0.0531
auxiliary peak (2)	$6.7724 \cdot 10^{-10}$	−1.4461	0.0366	$8.6815 \cdot 10^{-9}$	0.0311
auxiliary peak (3)	$1.0720 \cdot 10^{-9}$	0.0912	0.0376	$1.3397 \cdot 10^{-8}$	0.0319

C3 (background equation parameters a = $2.0262 \cdot 10^{-12}$; b = $3.6236 \cdot 10^{-12}$)

peak	a (area)	x_0 (position)	d_x (HWHM)	ampl	s
ZnT(1)	$1.8689 \cdot 10^{-10}$	−1.1228	0.1167	$7.5213 \cdot 10^{-10}$	0.0991
ZnT	$4.5816 \cdot 10^{-10}$	−0.9946	0.1115	$1.9304 \cdot 10^{-9}$	0.0947
ZnT(2)	$1.1006 \cdot 10^{-10}$	−0.8910	0.0622	$8.3138 \cdot 10^{-10}$	0.0528
ZnT(3)	$1.4047 \cdot 10^{-10}$	−0.7839	0.0674	$9.7825 \cdot 10^{-10}$	0.0573
ZnT(4)	$6.9008 \cdot 10^{-11}$	−0.6618	0.0556	$5.8303 \cdot 10^{-10}$	0.0472
MT(Cd)	$2.6357 \cdot 10^{-11}$	−0.5793	0.0424	$2.9181 \cdot 10^{-10}$	0.0360
MT(Pb)	$2.2158 \cdot 10^{-11}$	−0.3106	0.0587	$1.7740 \cdot 10^{-10}$	0.0498
MT(Cu)	$1.9121 \cdot 10^{-9}$	−0.0501	0.1390	$6.4636 \cdot 10^{-9}$	0.1180
auxiliary peak (1)	$4.8297 \cdot 10^{-9}$	−1.6788	0.0724	$3.1325 \cdot 10^{-8}$	0.0615
auxiliary peak (2)	$5.3906 \cdot 10^{-10}$	−1.5192	0.0762	$3.3245 \cdot 10^{-9}$	0.0647
auxiliary peak (3)	$1.1869 \cdot 10^{-9}$	0.1390	0.0707	$7.8843 \cdot 10^{-9}$	0.0601

Tables S2. List of individual peak parameters for hMT3 voltammograms (Fig S3. A1-C3).

General peak equation: $y(x) = \sqrt{\frac{\ln 2}{\pi}} \cdot \left(\frac{a}{d_x}\right) \cdot e^{\left(\frac{-\ln 2 \cdot (x-x_0)^2}{d_x^2}\right)}$.

Linear background correction equation: $y(x) = ax + b$.

A1 (background equation parameters a = $4.9848 \cdot 10^{-11}$; b = $7.2692 \cdot 10^{-10}$)

peak	a (area)	x_0 (position)	d_x (HWHM)	ampl	s
ZnT(1)	$4.4610 \cdot 10^{-12}$	−1.2270	0.0290	$7.2374 \cdot 10^{-11}$	0.0246
ZnT(2)	$7.6014 \cdot 10^{-12}$	−1.1716	0.0393	$9.0907 \cdot 10^{-11}$	0.0334
ZnT(3)	$5.1086 \cdot 10^{-11}$	−1.0749	0.0701	$3.4236 \cdot 10^{-10}$	0.0595
ZnT	$1.5030 \cdot 10^{-10}$	−0.9634	0.0601	$1.1741 \cdot 10^{-9}$	0.0511
ZnT(4)	$2.9935 \cdot 10^{-11}$	−0.8651	0.0586	$2.4007 \cdot 10^{-10}$	0.0497
MT(Cd)	$2.0292 \cdot 10^{-11}$	−0.6441	0.0398	$2.3942 \cdot 10^{-10}$	0.0338
MT(Pb)	$1.8198 \cdot 10^{-11}$	−0.4910	0.0702	$1.2180 \cdot 10^{-10}$	0.0596
MT(Cu)	$3.2700 \cdot 10^{-10}$	−0.2373	0.1078	$1.4242 \cdot 10^{-9}$	0.0916
MT(Cu(1))	$2.2941 \cdot 10^{-11}$	−0.1579	0.0361	$2.9819 \cdot 10^{-10}$	0.0307
auxiliary peak (1)	$3.3011 \cdot 10^{-10}$	−1.5228	0.0315	$4.9241 \cdot 10^{-9}$	0.0267
auxiliary peak (2)	$1.0763 \cdot 10^{-9}$	−1.5457	0.0784	$6.4485 \cdot 10^{-9}$	0.0666
auxiliary peak (3)	$7.3767 \cdot 10^{-10}$	0.0065	0.0179	$1.9356 \cdot 10^{-8}$	0.0152
auxiliary peak (4)	$1.1557 \cdot 10^{-8}$	0.1313	0.0872	$6.2263 \cdot 10^{-8}$	0.0740
auxiliary peak (5)	$7.6650 \cdot 10^{-11}$	−0.0857	0.0497	$7.2502 \cdot 10^{-10}$	0.0422

A2 (background equation parameters a = $5.7516 \cdot 10^{-11}$; b = $8.8065 \cdot 10^{-10}$)

peak	a (area)	x_0 (position)	d_x (HWHM)	ampl	s
ZnT(1)	$7.9052 \cdot 10^{-12}$	−1.2555	0.0375	$9.9011 \cdot 10^{-11}$	0.0319
ZnT(2)	$3.2388 \cdot 10^{-11}$	−1.1817	0.0487	$3.1210 \cdot 10^{-10}$	0.0414
ZnT(3)	$3.7448 \cdot 10^{-11}$	−1.1116	0.0540	$3.2580 \cdot 10^{-10}$	0.0459
ZnT	$2.0640 \cdot 10^{-10}$	−1.0016	0.0705	$1.3743 \cdot 10^{-9}$	0.0599
ZnT(4)	$9.0650 \cdot 10^{-11}$	−0.8928	0.0934	$4.5604 \cdot 10^{-10}$	0.0793
MT(Cd)	$1.6234 \cdot 10^{-11}$	−0.6381	0.0423	$1.8039 \cdot 10^{-10}$	0.0359
MT(Pb)	$1.6765 \cdot 10^{-11}$	−0.4697	0.0540	$1.4571 \cdot 10^{-10}$	0.0459
MT(Cu)	$5.1319 \cdot 10^{-10}$	−0.2210	0.1072	$2.2494 \cdot 10^{-9}$	0.0910
MT(Cu(1))	$5.7098 \cdot 10^{-11}$	−0.1202	0.0444	$6.0340 \cdot 10^{-10}$	0.0378
auxiliary peak (1)	$2.3109 \cdot 10^{-7}$	−1.8450	0.1400	$7.7532 \cdot 10^{-7}$	0.1189
auxiliary peak (2)	$2.4966 \cdot 10^{-10}$	−0.0465	0.0381	$3.0758 \cdot 10^{-9}$	0.0324
auxiliary peak (3)	$3.4951 \cdot 10^{-9}$	0.0461	0.0444	$3.6992 \cdot 10^{-8}$	0.0377

A3 (background equation parameters a = $-6.5421 \cdot 10^{-11}$; b = $8.9126 \cdot 10^{-10}$)

peak	a (area)	x_0 (position)	d_x (HWHM)	ampl	s
ZnT(1)	$1.3912 \cdot 10^{-11}$	−1.2448	0.0392	$1.6674 \cdot 10^{-10}$	0.0333
ZnT(2)	$6.0764 \cdot 10^{-12}$	−1.1985	0.0252	$1.1341 \cdot 10^{-10}$	0.0214
ZnT(3)	$4.6031 \cdot 10^{-11}$	−1.1499	0.0487	$4.4356 \cdot 10^{-10}$	0.0414
ZnT	$2.4761 \cdot 10^{-10}$	−1.0149	0.0722	$1.6105 \cdot 10^{-9}$	0.0613
ZnT(4)	$9.8017 \cdot 10^{-11}$	−0.8850	0.0915	$5.0297 \cdot 10^{-10}$	0.0777
MT(Cd)	$1.6916 \cdot 10^{-11}$	−0.6315	0.0453	$1.7533 \cdot 10^{-10}$	0.0385
MT(Pb)	$2.6737 \cdot 10^{-11}$	−0.4619	0.0589	$2.1329 \cdot 10^{-10}$	0.0500
MT(Cu)	$6.9118 \cdot 10^{-10}$	−0.2196	0.1103	$2.9443 \cdot 10^{-9}$	0.0937
MT(Cu(1))	$5.9624 \cdot 10^{-11}$	−0.1163	0.0444	$6.3028 \cdot 10^{-10}$	0.0377
auxiliary peak (1)	$6.5299 \cdot 10^{-9}$	−1.5998	0.0764	$4.0155 \cdot 10^{-8}$	0.0649
auxiliary peak (2)	$6.3815 \cdot 10^{-11}$	−1.4372	0.0455	$6.5811 \cdot 10^{-10}$	0.0387
auxiliary peak (3)	$4.0991 \cdot 10^{-10}$	−0.0309	0.0463	$4.1595 \cdot 10^{-9}$	0.0393
auxiliary peak (4)	$9.9279 \cdot 10^{-9}$	0.0735	0.0506	$9.2190 \cdot 10^{-8}$	0.0430

B1 (background equation parameters a = −9.7892·10^{-10}; b = 1.5117·10^{-10})

peak	a (area)	x_0 (position)	d_x (HWHM)	ampl	s
ZnT(1)	$1.3991\cdot10^{-11}$	−1.2236	0.0501	$1.3130\cdot10^{-10}$	0.0425
ZnT(2)	$9.2801\cdot10^{-12}$	−1.1684	0.0377	$1.1549\cdot10^{-10}$	0.0321
ZnT(3)	$5.2055\cdot10^{-11}$	−1.1001	0.0573	$4.2645\cdot10^{-10}$	0.0487
ZnT	$2.0452\cdot10^{-10}$	−0.9530	0.0939	$1.0235\cdot10^{-9}$	0.0797
ZnT(4)	$3.8566\cdot10^{-11}$	−0.7678	0.0700	$2.5865\cdot10^{-10}$	0.0595
MT(Cd)	$1.7148\cdot10^{-11}$	−0.4871	0.0498	$1.6164\cdot10^{-10}$	0.0423
MT(Pb)	$3.8806\cdot10^{-11}$	−0.3531	0.0672	$2.7119\cdot10^{-10}$	0.0571
MT(Cu)	$1.1050\cdot10^{-9}$	−0.1397	0.1179	$4.4023\cdot10^{-9}$	0.1001
MT(Cu(1))	$1.6996\cdot10^{-11}$	−0.1276	0.0516	$1.5486\cdot10^{-10}$	0.0438
auxiliary peak (1)	$3.9634\cdot10^{-9}$	−1.5725	0.0718	$2.5927\cdot10^{-8}$	0.0610
auxiliary peak (2)	$1.2401\cdot10^{-10}$	−1.4817	0.0266	$2.1872\cdot10^{-9}$	0.0226
auxiliary peak (3)	$2.3554\cdot10^{-10}$	−1.4449	0.0621	$1.7827\cdot10^{-9}$	0.0527
auxiliary peak (4)	$1.9811\cdot10^{-10}$	−0.0122	0.0311	$2.9970\cdot10^{-9}$	0.0264
auxiliary peak (5)	$7.6310\cdot10^{-10}$	0.0559	0.0411	$8.7223\cdot10^{-9}$	0.0349

B2 (background equation parameters a = −8.4822·10^{-11}; b = 8.9012·10^{-10})

peak	a (area)	x_0 (position)	d_x (HWHM)	ampl	s
ZnT(1)	$4.4571\cdot10^{-11}$	−1.2427	0.0512	$4.0866\cdot10^{-10}$	0.0435
ZnT(2)	$3.6942\cdot10^{-11}$	−1.1707	0.0430	$4.0362\cdot10^{-10}$	0.0365
ZnT(3)	$3.4846\cdot10^{-10}$	−1.0901	0.0962	$1.7011\cdot10^{-9}$	0.0817
ZnT	$1.2077\cdot10^{-10}$	−1.0744	0.0500	$1.1346\cdot10^{-9}$	0.0425
ZnT(4)	$3.9639\cdot10^{-10}$	−0.9312	0.1406	$1.3241\cdot10^{-9}$	0.1194
MT(Cd)	$8.0586\cdot10^{-11}$	−0.6610	0.0560	$6.7545\cdot10^{-10}$	0.0476
MT(Pb)	$1.2224\cdot10^{-10}$	−0.3866	0.1006	$5.7071\cdot10^{-10}$	0.0854
MT(Cu)	$1.5669\cdot10^{-9}$	−0.1646	0.1268	$5.8021\cdot10^{-9}$	0.1077
MT(Cu(1))	$1.3025\cdot10^{-10}$	−0.0879	0.0606	$1.0095\cdot10^{-9}$	0.0515
auxiliary peak (1)	$1.2969\cdot10^{-9}$	−1.6506	0.1172	$5.1993\cdot10^{-9}$	0.0995
auxiliary peak (2)	$2.9730\cdot10^{-11}$	−1.4936	0.0352	$3.9719\cdot10^{-10}$	0.0299
auxiliary peak (3)	$2.0673\cdot10^{-10}$	−0.0287	0.0366	$2.6500\cdot10^{-9}$	0.0311
auxiliary peak (4)	$1.4232\cdot10^{-9}$	0.0223	0.0434	$1.5414\cdot10^{-8}$	0.0368

B3 (background equation parameters a = 4.2638·10^{-10}; b = 1.7243·10^{-9})

peak	a (area)	x_0 (position)	d_x (HWHM)	ampl	s
ZnT(1)	$9.6486\cdot10^{-11}$	−1.2689	0.0589	$7.7005\cdot10^{-10}$	0.0500
ZnT(2)	$1.6605\cdot10^{-10}$	−1.1892	0.0500	$1.5600\cdot10^{-9}$	0.0425
ZnT(3)	$8.3975\cdot10^{-10}$	−1.0852	0.0696	$5.6702\cdot10^{-9}$	0.0591
ZnT	$7.5109\cdot10^{-10}$	−0.9468	0.0858	$4.1103\cdot10^{-9}$	0.0729
ZnT(4)	$1.8159\cdot10^{-10}$	−0.7996	0.0697	$1.2246\cdot10^{-9}$	0.0592
MT(Cd)	$1.3017\cdot10^{-10}$	−0.6600	0.0613	$9.9772\cdot10^{-10}$	0.0520
MT(Pb)	$1.6794\cdot10^{-10}$	−0.3400	0.0934	$8.4417\cdot10^{-10}$	0.0794
MT(Cu)	$3.1405\cdot10^{-9}$	−0.1163	0.1379	$1.0696\cdot10^{-8}$	0.1171
MT(Cu(1))	$1.2700\cdot10^{-10}$	−0.1305	0.0541	$1.1024\cdot10^{-9}$	0.0460
auxiliary peak (1)	$2.9746\cdot10^{-9}$	−1.7400	0.1112	$1.2567\cdot10^{-8}$	0.0944
auxiliary peak (2)	$9.3215\cdot10^{-11}$	−1.5529	0.0797	$5.4948\cdot10^{-10}$	0.0677
auxiliary peak (3)	$5.3469\cdot10^{-10}$	−0.0138	0.0366	$6.8543\cdot10^{-9}$	0.0311
auxiliary peak (4)	$1.8426\cdot10^{-9}$	0.0489	0.0428	$2.0202\cdot10^{-8}$	0.0364

C1 (background equation parameters a = $-2.9553\cdot10^{-9}$; b = $-1.3765\cdot10^{-9}$)

peak	a (area)	x_0 (position)	d_x (HWHM)	ampl	s
ZnT(1)	$1.8733\cdot10^{-12}$	−1.3660	0.0134	$6.5715\cdot10^{-11}$	0.0114
ZnT(2)	$2.9557\cdot10^{-11}$	−1.2683	0.0396	$3.5039\cdot10^{-10}$	0.0337
ZnT(3)	$6.3720\cdot10^{-11}$	−1.1594	0.1057	$2.8304\cdot10^{-10}$	0.0898
ZnT	$1.3200\cdot10^{-10}$	−1.0571	0.1008	$6.1516\cdot10^{-10}$	0.0856
ZnT(4)	$3.8006\cdot10^{-11}$	−0.8789	−0.8789	$2.8633\cdot10^{-10}$	0.0530
MT(Cd)	$1.6656\cdot10^{-12}$	−0.5750	0.0218	$3.5887\cdot10^{-11}$	0.0185
MT(Pb)	$1.5344\cdot10^{-10}$	−0.4607	0.1267	$5.6886\cdot10^{-10}$	0.1076
MT(Cu)	$1.8299\cdot10^{-9}$	−0.2144	0.1199	$7.1709\cdot10^{-9}$	0.1018
MT(Cu(1))	$1.2241\cdot10^{-10}$	−0.1147	0.0208	$2.7640\cdot10^{-9}$	0.0177
auxiliary peak (1)	$5.1899\cdot10^{-10}$	−1.5105	0.0203	$1.2021\cdot10^{-8}$	0.0172
auxiliary peak (2)	$1.0883\cdot10^{-10}$	−1.4739	0.0298	$1.7140\cdot10^{-9}$	0.0253
auxiliary peak (3)	$5.7382\cdot10^{-11}$	−1.4447	0.0519	$5.1972\cdot10^{-10}$	0.0440
auxiliary peak (4)	$1.0070\cdot10^{-9}$	0.0039	0.1074	$4.4020\cdot10^{-9}$	0.0913
auxiliary peak (5)	$1.4304\cdot10^{-9}$	0.0114	0.0233	$2.8847\cdot10^{-8}$	0.0198

C2 (background equation parameters a = $-3.0837\cdot10^{-9}$; b = $-1.3134\cdot10^{-9}$)

peak	a (area)	x_0 (position)	d_x (HWHM)	ampl	s
ZnT(1)	$8.0603\cdot10^{-11}$	−1.2787	0.0640	$5.9202\cdot10^{-10}$	0.0543
ZnT(2)	$3.2157\cdot10^{-10}$	−1.1683	0.0854	$1.7677\cdot10^{-9}$	0.0726
ZnT(3)	$1.6211\cdot10^{-10}$	−1.0574	0.0580	$1.3123\cdot10^{-9}$	0.0493
ZnT	$1.2098\cdot10^{-10}$	−0.9612	0.0669	$8.4949\cdot10^{-10}$	0.0568
ZnT(4)	$1.1737\cdot10^{-10}$	−0.8573	0.0995	$5.5418\cdot10^{-10}$	0.0845
MT(Cd)	$2.5031\cdot10^{-11}$	−0.6426	0.0296	$3.9705\cdot10^{-10}$	0.0252
MT(Pb)	$6.9703\cdot10^{-11}$	−0.5295	0.0958	$3.4158\cdot10^{-10}$	0.0814
MT(Cu)	$2.6589\cdot10^{-9}$	−0.2164	0.1433	$8.7159\cdot10^{-9}$	0.1217
MT(Cu(1))	$2.7612\cdot10^{-10}$	−0.1155	0.0361	$3.5971\cdot10^{-9}$	0.0306
auxiliary peak (1)	$3.5641\cdot10^{-10}$	−1.5413	0.0574	$2.9169\cdot10^{-9}$	0.0487
auxiliary peak (2)	$9.5658\cdot10^{-10}$	−1.5322	0.0303	$1.4843\cdot10^{-8}$	0.0257
auxiliary peak (3)	$3.9320\cdot10^{-10}$	−0.0542	0.0268	$6.8996\cdot10^{-9}$	0.0227
auxiliary peak (4)	$6.6322\cdot10^{-9}$	0.0301	0.0391	$7.9671\cdot10^{-8}$	0.0332

C3 (background equation parameters a = $-9.0901\cdot10^{-10}$; b = $7.8839\cdot10^{-10}$)

peak	a (area)	x_0 (position)	d_x (HWHM)	ampl	s
ZnT(1)	$8.9836\cdot10^{-11}$	−1.2564	0.0583	$7.2386\cdot10^{-10}$	0.0495
ZnT(2)	$5.0587\cdot10^{-10}$	−1.1234	0.0920	$2.5816\cdot10^{-9}$	0.0782
ZnT(3)	$7.4778\cdot10^{-10}$	−0.9703	0.0968	$3.6285\cdot10^{-9}$	0.0822
ZnT	$1.2322\cdot10^{-10}$	−0.8249	0.0652	$8.8766\cdot10^{-10}$	0.0554
ZnT(4)	$1.4954\cdot10^{-10}$	−0.6774	0.0686	$1.0240\cdot10^{-9}$	0.0583
MT(Cd)	$3.4283\cdot10^{-11}$	−0.6384	0.0278	$5.7878\cdot10^{-10}$	0.0236
MT(Pb)	$3.2518\cdot10^{-10}$	−0.2864	0.0796	$1.9189\cdot10^{-9}$	0.0676
MT(Cu)	$2.5249\cdot10^{-9}$	−0.1426	0.1144	$1.0368\cdot10^{-8}$	0.0972
MT(Cu(1))	$7.2017\cdot10^{-10}$	−0.0077	0.0298	$1.1334\cdot10^{-8}$	0.0253
auxiliary peak (1)	$4.7085\cdot10^{-8}$	−1.7350	0.1317	$1.6791\cdot10^{-7}$	0.1119
auxiliary peak (2)	$5.1912\cdot10^{-11}$	−0.0146	0.0032	$7.5298\cdot10^{-9}$	0.0028
auxiliary peak (3)	$8.9953\cdot10^{-10}$	0.0003	0.0120	$3.5290\cdot10^{-8}$	0.0102

Tables S3. List of individual peak parameters for hMT2a voltammograms (Fig S4. A1-C3).

General peak equation: $y(x) = \sqrt{\frac{\ln 2}{\pi}} \cdot \left(\frac{a}{d_x}\right) \cdot e^{\left(\frac{-\ln 2 \cdot (x-x_0)^2}{d_x^2}\right)}$.

Linear background correction equation: $y(x) = ax + b$.

A1 (background equation parameters a = $1.5204 \cdot 10^{-11}$; b = $4.6663 \cdot 10^{-12}$)

peak	a (area)	x_0 (position)	d_x (HWHM)	ampl	s
ZnT(1)	$3.5729 \cdot 10^{-10}$	−1.1285	0.0807	$2.0795 \cdot 10^{-9}$	0.0685
ZnT	$5.6507 \cdot 10^{-10}$	−1.0068	0.0693	$3.8304 \cdot 10^{-9}$	0.0589
ZnT(2)	$1.5741 \cdot 10^{-10}$	−0.9099	0.0531	$1.3923 \cdot 10^{-9}$	0.0451
ZnT(3)	$5.9685 \cdot 10^{-10}$	−0.8272	0.0945	$2.9659 \cdot 10^{-9}$	0.0803
ZnT(4)	$1.2309 \cdot 10^{-10}$	−0.6639	0.0723	$8.0010 \cdot 10^{-10}$	0.0614
MT(Pb)	$1.6709 \cdot 10^{-10}$	−0.2252	0.0702	$1.1186 \cdot 10^{-9}$	0.0596
MT(Cu)	$2.2560 \cdot 10^{-9}$	−0.0659	0.0846	$1.2521 \cdot 10^{-8}$	0.0719
auxiliary peak (1)	$2.3109 \cdot 10^{-7}$	−1.6897	0.0724	$1.4988 \cdot 10^{-6}$	0.0615
auxiliary peak (2)	$5.1029 \cdot 10^{-9}$	−1.6218	0.1272	$1.8847 \cdot 10^{-8}$	0.1080

A2 (background equation parameters a = $-1.1093 \cdot 10^{-10}$; b = $-1.0189 \cdot 10^{-10}$)

peak	a (area)	x_0 (position)	d_x (HWHM)	ampl	s
ZnT(1)	$6.5535 \cdot 10^{-10}$	−1.1698	0.0948	$3.2455 \cdot 10^{-9}$	0.0806
ZnT	$1.9137 \cdot 10^{-10}$	−1.0837	0.0547	$1.6447 \cdot 10^{-9}$	0.0464
ZnT(2)	$5.2753 \cdot 10^{-10}$	−0.9893	0.0763	$3.2482 \cdot 10^{-9}$	0.0648
ZnT(3)	$3.0478 \cdot 10^{-10}$	−0.8539	0.0689	$2.0766 \cdot 10^{-9}$	0.0586
ZnT(4)	$9.8137 \cdot 10^{-11}$	−0.7290	0.0626	$7.3588 \cdot 10^{-10}$	0.0532
MT(Pb)	$2.3702 \cdot 10^{-10}$	−0.3082	0.0941	$1.1828 \cdot 10^{-9}$	0.0799
MT(Cu)	$2.6214 \cdot 10^{-9}$	−0.1465	0.1015	$1.2135 \cdot 10^{-8}$	0.0862
auxiliary peak (1)	$2.3109 \cdot 10^{-7}$	−1.6897	0.0724	$1.4988 \cdot 10^{-6}$	0.0615
auxiliary peak (2)	$3.5106 \cdot 10^{-9}$	−1.5602	0.0605	$2.7259 \cdot 10^{-8}$	0.0514
auxiliary peak (3)	$1.3324 \cdot 10^{-9}$	0.0308	0.0584	$1.0709 \cdot 10^{-8}$	0.0496

A3 (background equation parameters a = $-6.3657 \cdot 10^{-10}$; b = $1.1631 \cdot 10^{-9}$)

peak	a (area)	x_0 (position)	d_x (HWHM)	ampl	s
ZnT(1)	$8.9645 \cdot 10^{-10}$	−1.1685	0.1123	$3.7484 \cdot 10^{-9}$	0.0954
ZnT	$2.4334 \cdot 10^{-10}$	−1.0800	0.0775	$1.4751 \cdot 10^{-9}$	0.0658
ZnT(2)	$1.0135 \cdot 10^{-9}$	−0.9474	0.1168	$4.0764 \cdot 10^{-9}$	0.0992
ZnT(3)	$1.3479 \cdot 10^{-10}$	−0.7782	0.0693	$9.1370 \cdot 10^{-10}$	0.0589
ZnT(4)	$6.5540 \cdot 10^{-11}$	−0.6639	0.0619	$4.9756 \cdot 10^{-10}$	0.0526
MT(Pb)	$3.1299 \cdot 10^{-10}$	−0.3107	0.0856	$1.7171 \cdot 10^{-9}$	0.0727
MT(Cu)	$3.3980 \cdot 10^{-9}$	−0.1474	0.0960	$1.6624 \cdot 10^{-8}$	0.0815
auxiliary peak (1)	$2.9244 \cdot 10^{-8}$	−1.7251	0.1146	$1.1990 \cdot 10^{-7}$	0.0973
auxiliary peak (2)	$6.4011 \cdot 10^{-10}$	−1.5129	0.0248	$1.2139 \cdot 10^{-8}$	0.0210
auxiliary peak (3)	$1.4188 \cdot 10^{-9}$	0.0351	0.0604	$1.1036 \cdot 10^{-8}$	0.0513

B1 (background equation parameters $a = -1.3066\cdot10^{-11}$; $b = -2.4314\cdot10^{-11}$)

peak	a (area)	x_0 (position)	d_x (HWHM)	ampl	s
ZnT(1)	$2.8147\cdot10^{-10}$	−1.1459	0.0800	$1.6534\cdot10^{-9}$	0.0679
ZnT	$6.9612\cdot10^{-10}$	−1.0144	0.0788	$4.1506\cdot10^{-9}$	0.0669
ZnT(2)	$4.3160\cdot10^{-10}$	−0.8888	0.0722	$2.8064\cdot10^{-9}$	0.0614
ZnT(3)	$3.3703\cdot10^{-10}$	−0.7785	0.0823	$1.9224\cdot10^{-9}$	0.0699
ZnT(4)	$1.4375\cdot10^{-10}$	−0.6369	0.0906	$7.4566\cdot10^{-10}$	0.0769
MT(Pb)	$2.2101\cdot10^{-10}$	−0.2133	0.0775	$1.3400\cdot10^{-9}$	0.0658
MT(Cu)	$2.2441\cdot10^{-9}$	−0.0647	0.0840	$1.2547\cdot10^{-8}$	0.0714
auxiliary peak (1)	$2.3109\cdot10^{-7}$	−1.6897	0.0724	$1.4988\cdot10^{-6}$	0.0615
auxiliary peak (2)	$5.2308\cdot10^{-9}$	−1.6251	0.1281	$1.9186\cdot10^{-8}$	0.1088

B2 (background equation parameters $a = 3.3533\cdot10^{-12}$; $b = 1.3382\cdot10^{-9}$)

peak	a (area)	x_0 (position)	d_x (HWHM)	ampl	s
ZnT(1)	$1.3117\cdot10^{-9}$	−1.0815	0.1295	$4.7584\cdot10^{-9}$	0.1100
ZnT	$5.7143\cdot10^{-10}$	−0.9911	0.0864	$3.1059\cdot10^{-9}$	0.0734
ZnT(2)	$6.0683\cdot10^{-10}$	−0.9017	0.0631	$4.5186\cdot10^{-9}$	0.0536
ZnT(3)	$4.5961\cdot10^{-10}$	−0.7846	0.0731	$2.9523\cdot10^{-9}$	0.0621
ZnT(4)	$1.8489\cdot10^{-10}$	−0.6467	0.0657	$1.3219\cdot10^{-9}$	0.0558
MT(Pb)	$4.1888\cdot10^{-10}$	−0.2581	0.0657	$2.9948\cdot10^{-9}$	0.0558
MT(Cu)	$4.4953\cdot10^{-9}$	−0.0698	0.1023	$2.0635\cdot10^{-8}$	0.0869
auxiliary peak (1)	$4.3346\cdot10^{-7}$	−1.6893	0.0641	$3.1743\cdot10^{-6}$	0.0545
auxiliary peak (2)	$1.2389\cdot10^{-10}$	−1.4891	0.0152	$3.8260\cdot10^{-9}$	0.0129
auxiliary peak (3)	$3.9174\cdot10^{-9}$	0.1343	0.0814	$2.2607\cdot10^{-8}$	0.0691

B3 (background equation parameters $a = 2.0672\cdot10^{-10}$; $b = 2.6186\cdot10^{-9}$)

peak	a (area)	x_0 (position)	d_x (HWHM)	ampl	s
ZnT(1)	$4.6503\cdot10^{-10}$	−1.2399	0.0776	$2.8163\cdot10^{-9}$	0.0659
ZnT	$1.6956\cdot10^{-9}$	−1.1088	0.0863	$9.2316\cdot10^{-9}$	0.0733
ZnT(2)	$2.2459\cdot10^{-9}$	−0.9855	0.0924	$1.1415\cdot10^{-8}$	0.0785
ZnT(3)	$1.6228\cdot10^{-9}$	−0.8561	0.1005	$7.5873\cdot10^{-9}$	0.0853
ZnT(4)	$1.7713\cdot10^{-10}$	−0.6860	0.0656	$1.2685\cdot10^{-9}$	0.0557
MT(Pb)	$4.8403\cdot10^{-10}$	−0.3165	0.0805	$2.8248\cdot10^{-9}$	0.0684
MT(Cu)	$1.0320\cdot10^{-8}$	−0.1150	0.1401	$3.4604\cdot10^{-8}$	0.1190
auxiliary peak (1)	$4.1086\cdot10^{-8}$	−1.6997	0.0724	$2.6648\cdot10^{-7}$	0.0615
auxiliary peak (2)	$5.5472\cdot10^{-10}$	−1.5159	0.0317	$8.2233\cdot10^{-9}$	0.0269
auxiliary peak (3)	$1.4640\cdot10^{-10}$	−0.0669	0.0311	$2.2136\cdot10^{-9}$	0.0264

C1 (background equation parameters $a = -2.6703\cdot10^{-9}$; $b = 1.5910\cdot10^{-9}$)

peak	a (area)	x_0 (position)	d_x (HWHM)	ampl	s
ZnT(1)	$3.4688\cdot10^{-10}$	−1.2171	0.0705	$2.3123\cdot10^{-9}$	0.0598
ZnT	$1.9229\cdot10^{-9}$	−1.1019	0.0879	$1.0271\cdot10^{-8}$	0.0747
ZnT(2)	$1.6347\cdot10^{-9}$	−0.9766	0.0876	$8.7671\cdot10^{-9}$	0.0744
ZnT(3)	$1.5056\cdot10^{-9}$	−0.8481	0.1167	$6.0594\cdot10^{-9}$	0.0991
ZnT(4)	$1.1794\cdot10^{-10}$	−0.6074	0.0814	$6.8060\cdot10^{-10}$	0.0691
MT(Pb)	$2.2583\cdot10^{-10}$	−0.3190	0.0888	$1.1949\cdot10^{-9}$	0.0754
MT(Cu)	$7.8246\cdot10^{-9}$	−0.1443	0.1102	$3.3359\cdot10^{-8}$	0.0936
auxiliary peak (1)	$1.7598\cdot10^{-7}$	−1.5825	0.0472	$1.7526\cdot10^{-6}$	0.0401
auxiliary peak (2)	$4.6203\cdot10^{-10}$	−1.4446	0.0435	$4.9938\cdot10^{-9}$	0.0369
auxiliary peak (3)	$4.1539\cdot10^{-10}$	−0.0586	0.0476	$4.0967\cdot10^{-9}$	0.0405
auxiliary peak (4)	$1.5772\cdot10^{-9}$	0.0254	0.0461	$1.6079\cdot10^{-8}$	0.0391

C2 (background equation parameters a = $-9.3880\cdot10^{-10}$; b = $2.4512\cdot10^{-9}$)

peak	a (area)	x_0 (position)	d_x (HWHM)	ampl	s
ZnT(1)	$3.5518\cdot10^{-10}$	−1.2526	0.0691	$2.4158\cdot10^{-9}$	0.0587
ZnT	$3.1496\cdot10^{-9}$	−1.0980	0.1063	$1.3924\cdot10^{-8}$	0.0902
ZnT(2)	$2.9633\cdot10^{-9}$	−0.9399	0.1047	$1.3289\cdot10^{-8}$	0.0890
ZnT(3)	$1.3322\cdot10^{-9}$	−0.7749	0.1102	$5.6796\cdot10^{-9}$	0.0936
ZnT(4)	$2.2589\cdot10^{-10}$	−0.5760	0.0696	$1.5240\cdot10^{-9}$	0.0591
MT(Pb)	$6.2219\cdot10^{-11}$	−0.4145	0.0495	$5.9000\cdot10^{-10}$	0.0421
MT(Cu)	$1.2022\cdot10^{-8}$	−0.1090	0.1285	$4.3948\cdot10^{-8}$	0.1091
auxiliary peak (1)	$1.7082\cdot10^{-8}$	−1.6479	0.1195	$6.7135\cdot10^{-8}$	0.1015
auxiliary peak (2)	$3.3159\cdot10^{-11}$	−1.4800	0.0055	$2.8203\cdot10^{-9}$	0.0047
auxiliary peak (3)	$1.9806\cdot10^{-9}$	0.1214	0.0692	$1.3452\cdot10^{-8}$	0.0587

C3 (background equation parameters a = $-2.8908\cdot10^{-9}$; b = $1.3964\cdot10^{-9}$)

peak	a (area)	x_0 (position)	d_x (HWHM)	ampl	s
ZnT(1)	$2.3320\cdot10^{-10}$	−1.2159	0.0612	$1.7896\cdot10^{-9}$	0.0520
ZnT	$2.8917\cdot10^{-9}$	−1.0739	0.1093	$1.2427\cdot10^{-8}$	0.0928
ZnT(2)	$1.7485\cdot10^{-9}$	−0.9162	0.0972	$8.4452\cdot10^{-9}$	0.0826
ZnT(3)	$4.9953\cdot10^{-10}$	−0.7585	0.0845	$2.7761\cdot10^{-9}$	0.0718
ZnT(4)	$1.1385\cdot10^{-10}$	−0.5950	0.0610	$8.7702\cdot10^{-10}$	0.0518
MT(Pb)	$5.8961\cdot10^{-11}$	−0.4020	0.0600	$4.6159\cdot10^{-10}$	0.0510
MT(Cu)	$8.7893\cdot10^{-9}$	−0.1351	0.1205	$3.4274\cdot10^{-8}$	0.1023
auxiliary peak (1)	$4.9421\cdot10^{-11}$	−1.4791	0.0031	$7.5150\cdot10^{-9}$	0.0026
auxiliary peak (2)	$4.6186\cdot10^{-10}$	−1.4728	0.0121	$1.7984\cdot10^{-8}$	0.0102
auxiliary peak (3)	$1.3552\cdot10^{-9}$	−1.4809	0.0526	$1.2098\cdot10^{-8}$	0.0447
auxiliary peak (4)	$1.1886\cdot10^{-9}$	−1.4930	0.0148	$3.7749\cdot10^{-8}$	0.0126
auxiliary peak (5)	$2.1419\cdot10^{-9}$	0.0506	0.0600	$1.6768\cdot10^{-8}$	0.0510
